# Drug-induced Liver Disease in Patients with Diabetes Mellitus

**DOI:** 10.5005/jp-journals-10018-1140

**Published:** 2016-07-09

**Authors:** Klyarytskaya Iryna, Maksymova Helen, Stilidi Elena

**Affiliations:** 1 Department of Therapy and Family Medicine, Faculty of Postgraduate Education, Medical Academy, SI Georgievsky, Simferopol Crimea, Russian Federation

**Keywords:** Diabetes mellitus, Drug-induced liver disease, Fibrosis, Hepatotoxicity, Methacetin breath test, Risk factors.

## Abstract

The study presented here was accomplished to assess the course of drug-induced liver diseases in patient’s rheumatoid arthritis receiving long-term methotrexate therapy. Diabetes mellitus was revealed as the most significant risk factor. The combination of diabetes mellitus with other risk factors (female sex) resulted in increased hepatic fibrosis, degree of hepatic encephalopathy and reduction of hepatic functions. The effectiveness and safety of ursodeoxycholic acid and cytolytic type-with S-Adenosyl methionine was also evaluated.

**Abbreviations:**

13C-MBT: 13C-methacetin breath test; ALT: alanine aminotransferase; AP: alkaline phosphatase; AST: aspartic transaminase; DILD: drug-induced liver disease; DM: diabetes mellitus; HE: hepatic encephalopathy; HFM: hepatic functional mass; SAMe: S-Adenosyl methionine; UDCA: ursodeoxycholic acid.

**How to cite this article:**

Iryna K, Helen M, Elena S. Drug-induced Liver Disease in Patients with Diabetes Mellitus. Euroasian J Hepato-Gastroenterol 2015;5(2):83-86.

## INTRODUCTION

The clinical course of drug-induced liver disease (DILD) is still unpredictable. Drug-induced liver disease is a complex clinical problem because the spectrums of clinical and morphological variants are extremely diverse; the diagnosis is made by means of exclusion and there are no clear principles of therapy, except withdrawal of the drug.

Although the physicians are aware of the possibility of hepatotoxic reactions to a variety of drugs, diagnosis is rarely formulated in clinical practice. Thus, in 2 to 5% of patients hospitalized with jaundice, DILD is revealed later. From 15 to 30% of cases of fulminant hepatic failure, and about 40% of cases of acute hepatitis in patients older than 40 years is associated with drug usage.^1^ In economically developed countries, DILD represents an important factor in overall morbidity and mortality. The true incidence of DILD is unknown because of lack of considerable numbers of pharmacoepidemiological studies.

Advances of modern chemotherapy have been successful in treating many malignant neoplasms that were fatal previously. Higher efficiency of treatment is achieved by intensification of chemotherapy regimens. However, all sorts of cytotoxic agents used for treatment of malignancies are capable of inducing hepatotoxicity.^2^ Withdrawal of causative agent is sufficient for the regres -sion of lesions in most cases of acute DILD. However, abolition of hepatotoxic drugs in case of chemotherapy in cancer patients is associated with immediate or delayed danger to the patient.^3^ Several factors (female gender, obesity, diabetes and smoking) increase the risk of DILD.^1^ The most significant risk factors are dose, duration of treatment, and drug concentration in the blood.^4^ However, history of concomitant illness can also play significant role during inducing DILD. Studies have shown that the risk of DILD, in particular the risk of fibrosis, is increased in patients with diabetes mellitus (DM).^5^

## MATERIALS AND METHODS

A total of 88 patients with rheumatoid arthritis receiving long-term methotrexate therapy were enrolled in this study. Depending on the risk factors, patients were divided into four groups (each containing 22 patients); group 1: women with concomitant DM; group 2: men with DM; group 3: patients with obesity (BMI >30 kg/m^2^) of both genders and group 4: patients of both genders, who smoked > 20 cigarettes per day.

The diagnosis of DILD was based on data of laboratory findings [More than 2 times elevation of alanine aminotransferase (ALT) and/or alkaline phosphatase (ALP)] and exclusion of other causes of liver disease (viral, autoimmune, hereditary), as well as alcoholic and non-alcoholic fatty liver disease.

Inclusion criteria included age over 18 years, patients with DM, increased ALT or ALP, exclusion of other causes of liver diseases; negative for markers of hepatitis B virus, hepatitis C virus, hepatitis D virus, and autoimmune hepatitis. Exclusion criteria included history of alcohol consumption, history of acute hepatitis during last 12 months, presence of concomitant hepatic decompensation, pregnancy and lactation.

Type of DILD was determined according to the degree of ALT and ALP elevation (Table 1).

All patients were checked for demographic data and medical history. Also, physical examination and biochemical test were accomplished in all patients. Viral markers and markers of autoimmunity were evaluated. Blood test for markers of fibrosis included alpha-2-macroglobulin, apolipoprotein A1, and haptoglobin. Critical flicker frequency test and ^13^C-methacetin breath test were also done.

During history-taking we paid special attention on possible causes of increased levels of ALT and ALP, history of alcohol intake and previous illnesses. Clinical and laboratory examination included determination of fasting glucose level, glucose tolerance test, blood biochemical examination (determination of bilirubin and its fractions, and the levels of AST, ALT and ALP.

The degree of hepatic encephalopathy (HE) was determined with the help of critical flicker frequency test with Hepatonorm^TM^ Analyser on 0 and 8 weeks of treatment. Flicker frequency analyzer causes intrafoveal light stimulus with certain light impulses of determined wavelength and brightness, which gradually reduces from 60 Hz. Intrafoveal stimuli was guaranteed by concave-convex lens system that leads the eye to the accommodation of the virtual picture of the light source. With this method, the frequency of red light, which was originally generated as a high frequency pulse (60 Hz), creates in a patient a sense of constant light, gradually decreases to flicker. The patient must note the change by keystroke. Critical flicker frequencies were measured several times after the stage of practice and the average of 8 to 9 measurements were calculated. This mean value is called the critical flicker frequency and used for assess the severity of HE. Flickering is usually determined at the values more than 42 Hz. In patients with minimal hepatic encephalopathy it is below 39 Hz (the critical value), in I grade of HE - for values 35.9-32 Hz, in II grade of HE - for values 31.9 to 28 Hz, in III grade of HE - values below 27.9 Hz.

^13^C-methacetin breath test (^13^C-MBT) was used to determine the mass of functioning hepatocytes.^6^ The principle of the method is that the non-radioactive labeled ^13^C-methacetin (a derivative of phenacetin) is exposed to enzymatic demethylation and decarboxylation with microsomal cytochrome P450 in the liver. The end product of metabolism of ^13^C-methacetin is ^13^CO_2_, the intensity of selection of which through the lungs gives possibility to indicate the functional status of the microsomal enzyme systems of hepatocytes. During the test, 10 respiratory samples were received: initial - before taking the test breakfast (75 mg ^13^C-methacetin dissolved in 200 ml of tea without sugar), 6 more samples during the first hour (one sample every 10 minutes) and three - during the second hour (one sample every 20 minutes). Analysis of samples was performed on an infrared spectrometer IRIS. Table 2 shows the performance of normal and pathological ^13^CO_2 _total concentration and its relation to hepatic functional mass (HFM). The sensitivity and specificity is over 90%.

From the point of view of evidence-based medicine, one of the most effective drugs for the treatment of drug-induced liver disease is ursodeoxycholic acid (UDCA), as they have a broad spectrum of action. Patients with cholestatic and mixed variant of DILD had been receiving UDCA 10 mg/kg once a day for 2 months and those with cytolytic variant of DILD had been receiving S-Adenosyl methionine (SAMe) 400 mg 3 times per day for 2 months.

**Table 1:** **Table 1:** Type of drug-induced liver disease

*Type*		*ALT*		*ALP*		*ALT/ALP*	
Cytolytic type		> 2		Normal		> 5	
Cholestatic type		Normal		> 2		< 2	
Mixed type		> 2		> 2		2-5	

**Table 2:** **Table 2:** Evaluation of ^13^C-methacetin breath test results

The total concentration of ^13^CO_2_ to the 120th minute, %		interpretation of results	
> 35%		Stimulated liver function	
20-35%		Normal liver function, HFM 100%	
10-20%		Moderate decrease of liver detoxification function without cirrhotic changes, HFM 50 to 100%	
2-10%		Marked reduction of liver detoxification function with cirrhotic changes, HFM 20 to 50%	
< 2%		Severe reduced function of the liver with cirrhotic changes, HFM < 20%	

## RESULTS AND DISCUSSION

**Graph 1:**
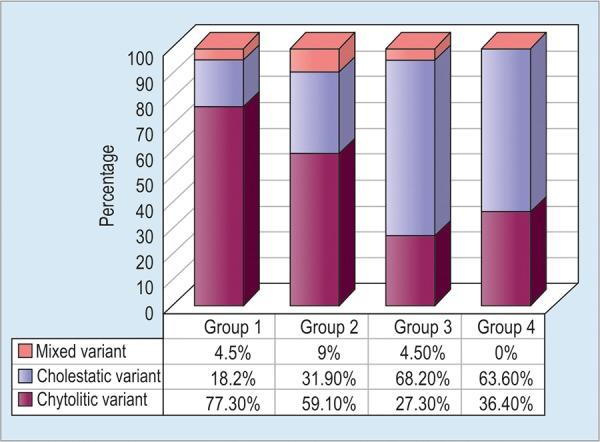
Distribution of DILD types in groups before treatment

Cytolytic variant of DILD was diagnosed more often in patients with DM, especially among women (77.3% in group 1 and 59.1% in group 2, p < 0.05). On the other hand, cholestatic variant of DILD was dominant in group 3 (68.2%) (Graph 1).

**Graph 2:**
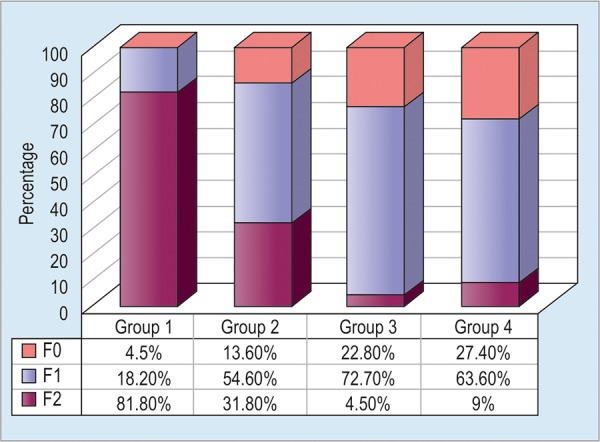
The distribution of fibrosis in groups before treatment

Serum markers of fibrosis, stage of fibrosis was also higher in group 1 (Graph 2).

According to the results of ^13^C-methacetin breath test, moderate decrease of hepatic functional mass was observed in most patients with DM (77.3% in group 1 and 68.2% in group 2) (Table 3).

**Graph 3:**
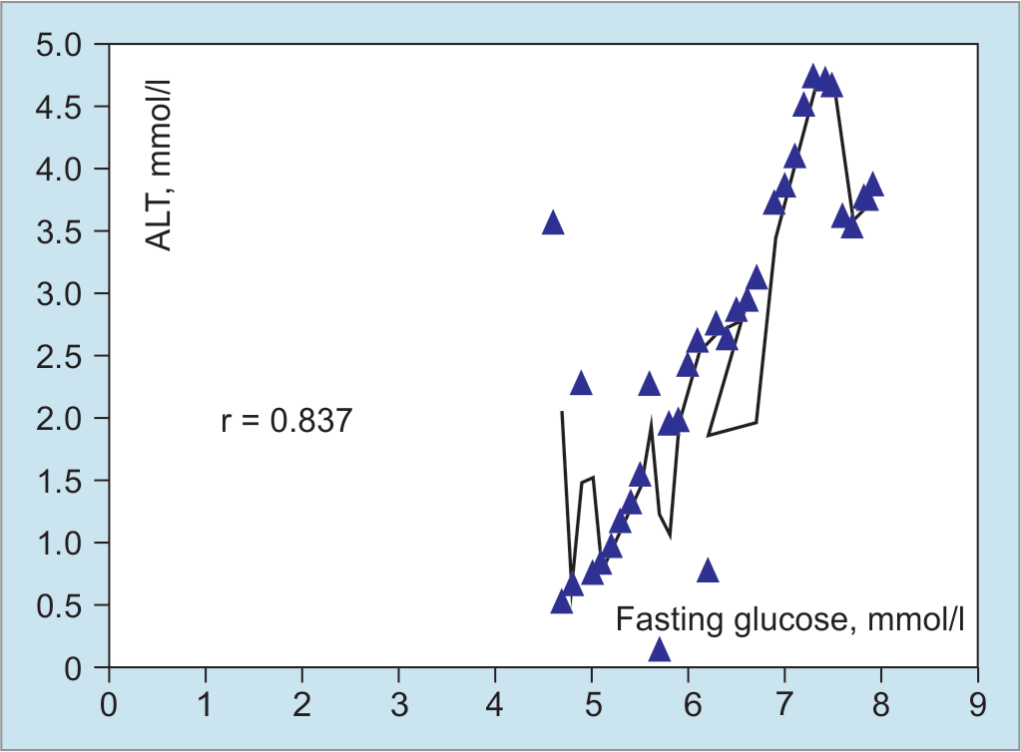
Correlation between levels of fasting glucose and ALT

**Graph 4:**
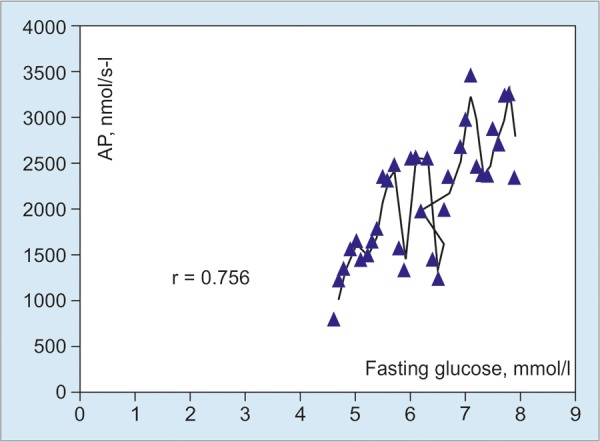
Correlation between levels of fasting glucose and ALP

Correlation and statistical analysis revealed that the level of fasting glucose closely positively correlated with both levels of ALT (r = 0.837) and ALP (r = 0.756) and did not correlate with the level of AST (Graphs 3 and 4).

**Graph 5:**
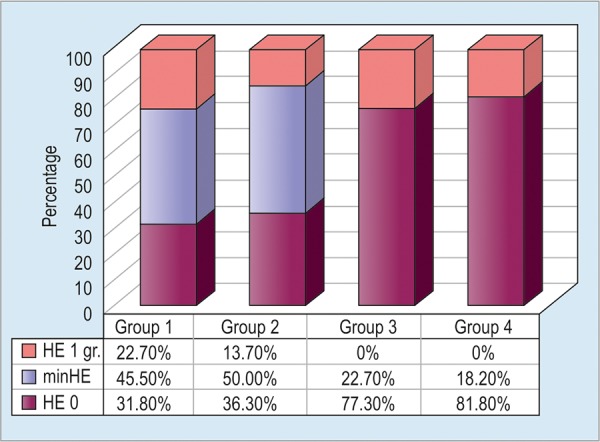
Distribution of HE grades in groups before treatment

Critical flicker frequency test indicate that the degree of HE was higher in group 1 (Graph 5).

Evaluation of treatment effectiveness was carried out on 8th week. The most effective treatment was significantly higher in groups without DM. Normalization of ALT and ALP was observed in 86.4 and 77.3% of patients in Groups 3 and 4, respectively).

**Graph 6:**
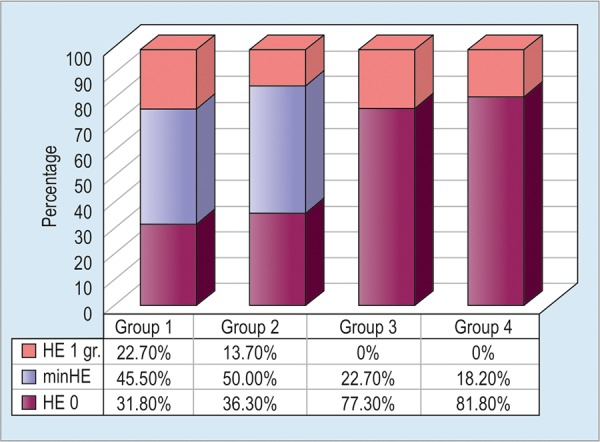
Distribution of HE grades in groups after treatment

Normal results of critical flicker frequency test and, accordingly, absence of HE, have also been registered in the majority of patients from the groups 3 and 4 (Graph 6).

Adherence to treatment and drug tolerability were evaluated in all patients during treatment on the basis of complaints and physical examination. There were no significant side-effects, due to which the patients were excluded from the study. All side-effects were minimal and did not require treatment cancellation. Increased stool was observed in 2 patients who had been receiving UDCA. Three patients complained of nausea, 1 patient for headaches receiving SAMe.

**Table 3:** **Table 3:** Results of ^13^C-methacetin breath test in groups before treatment

		*Normal* *liver* *function*				*Moderate* *decrease of liver* *detoxification* *function*				*Marked* *reduction of liver* *detoxification* *function*			
*Groups*		*n*		*%*		*n*		*%*		*n*		*%*	
1		1		4.5		17		77.3		4		18.2	
2		4		18.2		15		68.2		3		13.6	
3		11		50		10		45.5		1		4.5	
4		12		54.5		10		45.5		0		0	

Thus, the study revealed that worse treatment group consisted of patients with a combination of DILD and DM. Treatment for these patients is a complex task that requires a comprehensive approach involving gastroenterologists, endocrinologists and rheumatologists.
